# Induction of trained immunity in myeloid cells and their progenitors from thyroid cancer patients

**DOI:** 10.3389/fimmu.2025.1706496

**Published:** 2025-12-17

**Authors:** Pepijn van Houten, Prashant Changoer, Liesbeth van Emst, Han J. Bonenkamp, Johannes H. W. de Wilt, Willemijn Hobo, Manita E. J. Bremmers, Mieke W. H. Roeven, Janneke E. W. Walraven, Petronella B. Ottevanger, Willem J. M. Mulder, Mihai G. Netea, Martin Jaeger, Romana T. Netea-Maier

**Affiliations:** 1 Department of Internal Medicine, Division of Endocrinology, Radboud University Medical Center, Nijmegen, Netherlands; 2 Department of Surgery, Radboud University Medical Center, Nijmegen, Netherlands; 3 Department of Laboratory Medicine, Laboratory of Hematology, Radboud University Medical Center, Nijmegen, Netherlands; 4 Department of Hematology, Radboud University Medical Center, Nijmegen, Netherlands; 5 Department of Medical Oncology, Radboud University Medical Center, Nijmegen, Netherlands; 6 Laboratory of Chemical Biology, Department of Biomedical Engineering, Eindhoven University of Technology, Eindhoven, Netherlands; 7 Institute for Complex Molecular Systems (ICMS), Eindhoven University of Technology, Eindhoven, Netherlands; 8 Department of Internal Medicine, Radboud Community for Infectious Diseases (RCI), Radboud University Medical Center, Nijmegen, Netherlands; 9 Department of Immunology and Metabolism, Life and Medical Sciences Institute, University of Bonn, Bonn, Germany; 10 Research Center for Functional Genomics, Biomedicine and Translation Medicine, Iuliu Hatieganu University of Medicine and Pharmacy, Cluj-Napoca, Romania

**Keywords:** innate immunity, macrophages, monocytes, non-medullary thyroid carcinoma, trained immunity

## Abstract

**Background:**

The prognosis of patients with cancer in which tumor-associated macrophages with a pro-tumoral phenotype are abundant is poor. This includes aggressive forms of non-medullary thyroid carcinoma (NMTC). Trained immunity describes long-term epigenetic and metabolic reprogramming in innate immune cells and their bone marrow progenitors, leading to improved responsiveness, which is currently being explored as a potential new treatment approach in cancer. We aimed to assess whether trained immunity can be induced in myeloid cells of patients with NMTC to enhance the anti-tumor immune response, and whether this effect is tumor-specific or can be elicited in other forms of cancers sharing the immune-mediated pathophysiology, such as colorectal carcinoma (CRC).

**Methods:**

Peripheral blood and bone marrow were obtained from 53 NMTC patients (39 differentiated and 14 poorly differentiated/anaplastic NMTC) and 13 healthy controls. Peripheral monocytes and bone marrow progenitors were isolated *ex vivo*, and trained immunity was induced using different stimuli. Cytokine production upon restimulation and expression of cell membrane activation markers were used as biomarkers of cellular activation. In addition, trained immunity was assessed in peripheral monocytes from seven CRC patients.

**Results:**

Training of circulating monocytes with β-glucan or interleukin-4 resulted in amplified cytokine production upon restimulation, a hallmark of trained immunity responses. Fold changes of increase in cytokine production were comparable between the NMTC subtypes, CRC, and healthy controls. Flow cytometry showed that training of bone marrow progenitors resulted in macrophages with lower CD206 and CD163 and higher CD86 expression, a profile associated with a less immunosuppressive and more anti-tumoral phenotype.

**Conclusion:**

*Ex vivo* training of monocytes and bone marrow progenitors from patients with NMTC and CRC results in macrophages with increased proinflammatory cytokine production and differentiation toward an anti-tumoral phenotype. This suggests that trained immunity may be exploited as a potential novel treatment strategy for cancer.

## Introduction

The presence of an immunosuppressive tumor microenvironment (TME), and especially tumor-associated macrophages (TAMs), as a major component with inflammatory and lymphocyte-suppressive effects, is considered one of the main factors indicating poor prognosis in solid tumors (1). TAMs derive from tissue-resident macrophages or bone marrow-derived circulating monocytes: they inhibit cytotoxic T lymphocytes, while in parallel, they promote tumor cell proliferation, angiogenesis, and metastasis by secreting several pro-tumoral factors such as cytokines, chemokines, and growth factors (1). Furthermore, TAMs enhance immunosuppression in the TME by recruiting other immunosuppressing immune cells, such as regulatory T lymphocytes, which further facilitates tumor cell survival (2). Moreover, TAMs diminish the effectiveness of cancer immunotherapies such as the recently introduced immune checkpoint inhibitors (ICIs) (3). ICI treatment aims to enable T lymphocytes to effectively attack tumor cells. Since macrophages are highly plastic cells, reprogramming of TAMs toward a more anti-tumoral phenotype has been hypothesized to represent an exciting new treatment strategy, either alone or in combination with ICIs (4, 5).

Aggressive forms of non-medullary thyroid carcinoma (NMTC), especially anaplastic NMTC (ATC), are typically characterized by an abundance of TAMs in the tumor microenvironment, which negatively correlates with survival (6–8). In contrast with the most prevalent NMTC subtype, differentiated NMTC (DTC), which can mostly be treated with a combination of surgery and radioiodine treatment, resulting in an excellent prognosis (9), the prognosis worsens when the patients develop local recurrences or metastases that are refractory to radioiodine treatment (RAIR-DTC) (10), or when patients develop the rare undifferentiated and very aggressive ATC, mirrored by a median survival of only a few months (11). For the latter categories, treatment options are very limited. Importantly, TAMs have been involved in the pathogenesis of other malignant tumors as well. One illustrative example is colorectal carcinoma (CRC), which is associated with a median 5-year survival of less than 20% when patients present with or develop distant metastases (12, 13).

We have previously shown that both circulating innate immune cells and bone marrow progenitors from NMTC patients show functional and transcriptional changes compared to healthy controls and patients with benign thyroid tumors (14). This suggests that the rewiring of myeloid cells and their progenitors toward an immunosuppressive phenotype is already initiated before infiltration into the TME. Consequently, it may be hypothesized that myeloid cell reprogramming toward an anti-tumoral phenotype could provide beneficial effects in patients with cancer, such as NMTC and CRC. One recently emerging mechanism to potentially reverse the pro-tumoral phenotype of myeloid cells is represented by the induction of a *de facto* innate immune memory called “trained immunity” (15).

Trained immunity is the immune process through which certain endogenous or exogenous stimuli induce long-term metabolic and epigenetic rewiring of innate immune cells, resulting in an elevated antigen-agnostic immune response upon encounter with a secondary stimulus (15). In the last decade, several exogenous and endogenous stimuli have been described that can induce trained immunity (16–18). Notably, the effects of trained immunity last longer (months) than the lifespan of monocytes in circulation (days). This could be explained by the fact that after *in vivo* challenges, trained immunity is induced not only in circulating monocytes but also in hematopoietic stem and progenitor cells (19, 20). Due to the capacity to remodel and enhance the function of myeloid cells and their progenitors, the induction of trained immunity has been proposed as a potential novel approach for cancer immunotherapy (21). Moreover, trained myeloid cells have an increased ability to activate T cells (22). Indeed, several experimental studies have shown anti-tumoral effects induced by trained immunity through different stimuli such as β-glucan, influenza, and muramyl dipeptide, resulting in reduced tumor growth and improved survival (23–27), with some of these studies indicating a synergistic effect when the trained immunity-inducing agent was co-administered with immune checkpoint inhibitors (23, 25). Interestingly, these studies used different stimuli to induce trained immunity in animal models with different tumor types, underlining the wide range of cancers in which trained immunity could be explored as potential treatment. Moreover, the anti-tumoral effect of trained immunity was preserved after bone marrow transplantation from trained mice to naïve mice, emphasizing the role of bone marrow progenitors in the long-term persistence of trained immunity and the phenotype of tumor-infiltrating macrophages (23, 26, 27).

The induction of trained immunity in humans has mainly been demonstrated in patients with bladder cancer, in which treatment with intravesical Bacillus Calmette–Guérin (BCG) is an established immunotherapy (28). The induction of trained immunity by intravesical BCG has been demonstrated to be associated with a favorable outcome in these patients, supporting the concept of trained immunity-based immunotherapy in cancer (29). Unfortunately, the use of BCG, a live microorganism, in malignancies beyond bladder cancer is limited due to potential severe complications such as systemic disseminated BCG-osis. Therefore, other methods for trained immunity induction should be developed for applications in other cancers.

In the current study, based on the previous finding that myeloid cells from NMTC patients showed decreased cytokine production capacity and transcriptional reprogramming at the level of bone marrow progenitors, we aimed to investigate whether trained immunity can be induced *ex vivo* in circulating monocytes and bone marrow progenitors from patients with different histological subtypes of NMTC, compared with healthy controls (14). Next, we assessed which factors were associated with the induction of trained immunity. To investigate whether this effect is tumor-specific or can be elicited in other cancers sharing the immune-mediated pathophysiology, we assessed the induction of trained immunity in circulating monocytes from CRC patients. If successful, trained immunity induction either by *in vivo* administration of trained immunity inducers or by *ex vivo* training of myeloid cells and their progenitors, followed by their administration to patients, could be envisaged as a new form of immunotherapy in cancer. Furthermore, assessing the *ex vivo* trainability of the circulating myeloid cells could serve as a diagnostic test to predict who may benefit from adjuvant *in vivo* or *ex vivo* training and potentially to select the optimal training agonist in the context of precision medicine.

## Results

### Baseline characteristics

Peripheral blood was drawn from 13 healthy controls, 22 DTC patients, 14 RAIR-DTC patients, and 14 ATC/poorly differentiated thyroid carcinoma (PDTC) patients. In addition, blood was drawn from seven patients with CRC. A flowchart describing the assessment for eligibility and reasons for exclusions is shown in **Supplementary Figure S1**. Baseline and demographic characteristics of the participants are shown in **Table 1**. Healthy controls and DTC patients were younger and more frequently male than RAIR-DTC and ATC/PDTC patients. CRC patients were also older than healthy controls. Bone marrow aspirates were available from 13 NMTC patients (12 DTC and one RAIR-DTC; median age, 54 years; interquartile range (IQR), 47–72 years) and three healthy stem cell donors (two women aged 36 and 49 years, and one man aged 39 years).

**Table 1:** Baseline characteristics of the different subgroups.

	Healthy controls (*n* = 13)	DTC (*n* = 22)	RAIR-DTC (*n* = 14)	ATC/PDTC (*n* = 14)	CRC (*n* = 7)
Sex (male/female)	7/6	17/5	5/9	2/12	5/2
Age [median years (IQR)]	53 (31.5–63.5)	48 (38.75–60.25)	66 (62.25–76)	67 (62.75–74.25)	70 (62–74)
Subtype of DTC (*n*)	N/A			N/A	N/A
- PTC		21	8		
- FTC		0	2		
- OTC		1	4		
Subtype of CRC (*n*)					
- Colon					4
- Rectal					3
Previous treatment (*n*)	N/A				
- Surgery		0	13	5	4
- Radiotherapy		0	3	0	4
- Systemic therapy		0	0	2	4
Cervical lymph node metastases (*n*)	N/A	21	3	7	N/A
Distant metastases (*n)*	N/A				
- Lymph nodes		0	6	4	2
- Lungs		3	13	8	1
- Liver		0	1	0	1
- Bones		1	3	3	0
- Other		0	0	2	3

IQR, interquartile range; N/A, not applicable; DTC, differentiated thyroid carcinoma; RAIR, radioiodine refractory; PDTC, poorly differentiated thyroid carcinoma; ATC, anaplastic thyroid carcinoma; PTC, papillary thyroid carcinoma; FTC, follicular thyroid carcinoma; OTC, oncocytic thyroid carcinoma; CRC, colorectal carcinoma.

### Trained immunity induction in circulating monocytes isolated from patients with NMTC and CRC

**Figure 1:**
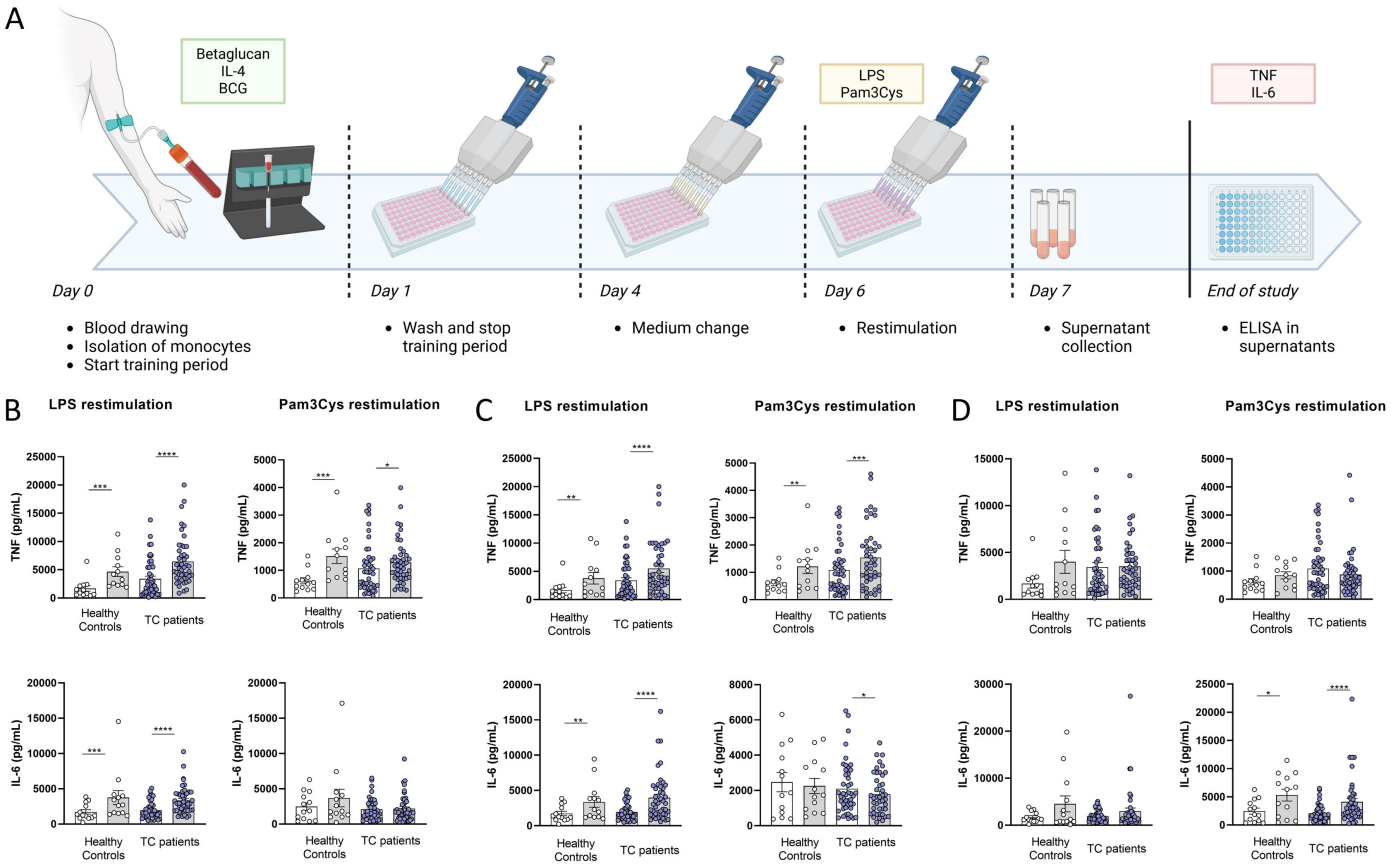
**(A)** Overview of the protocol for training of monocytes. Production of TNF and IL-6 after restimulation with LPS or Pam3Cys after training of circulating monocytes with IL-4 **(B)**, β-glucan **(C)**, or BCG **(D)** (gray bars) compared to untrained controls (white bars). *p < 0.05, **p < 0.01, ***p < 0.001, and ****p < 0.0001. LPS, lipopolysaccharide; BCG, Bacillus Calmette–Guérin.

To assess whether monocytes from NMTC patients can be trained, we exposed cells to β-glucan, IL-4, or BCG (**Figure 1**), which are stimuli known to result in training through different pathways (dectin-1, IL-4-receptor, and NOD2 receptor, respectively). As read-outs for training, we assessed the production of TNF and IL-6 upon stimulation with Toll-like receptor (TLR) 4 and TLR2 agonists (*Escherichia coli*-derived lipopolysaccharide (LPS) and Pam3Cys-Ser-(Lys)4 (Pam3Cys), respectively). Training with β-glucan or IL-4 resulted in higher concentrations of TNF after restimulation with LPS or Pam3Cys and higher concentrations of IL-6 after LPS restimulation, both in NMTC patients and in healthy controls. In contrast, Pam3Cys restimulation of IL-6 production was not altered after β-glucan training. IL-4 training resulted in lower production of Pam3Cys restimulated IL-6. BCG training resulted in elevated IL-6 production after Pam3Cys restimulation but did not alter TNF production. These results indicate that trained immunity can be induced in circulating monocytes from NMTC patients, albeit with different phenotypes depending on the trained immunity inducer.

**Figure 2:**
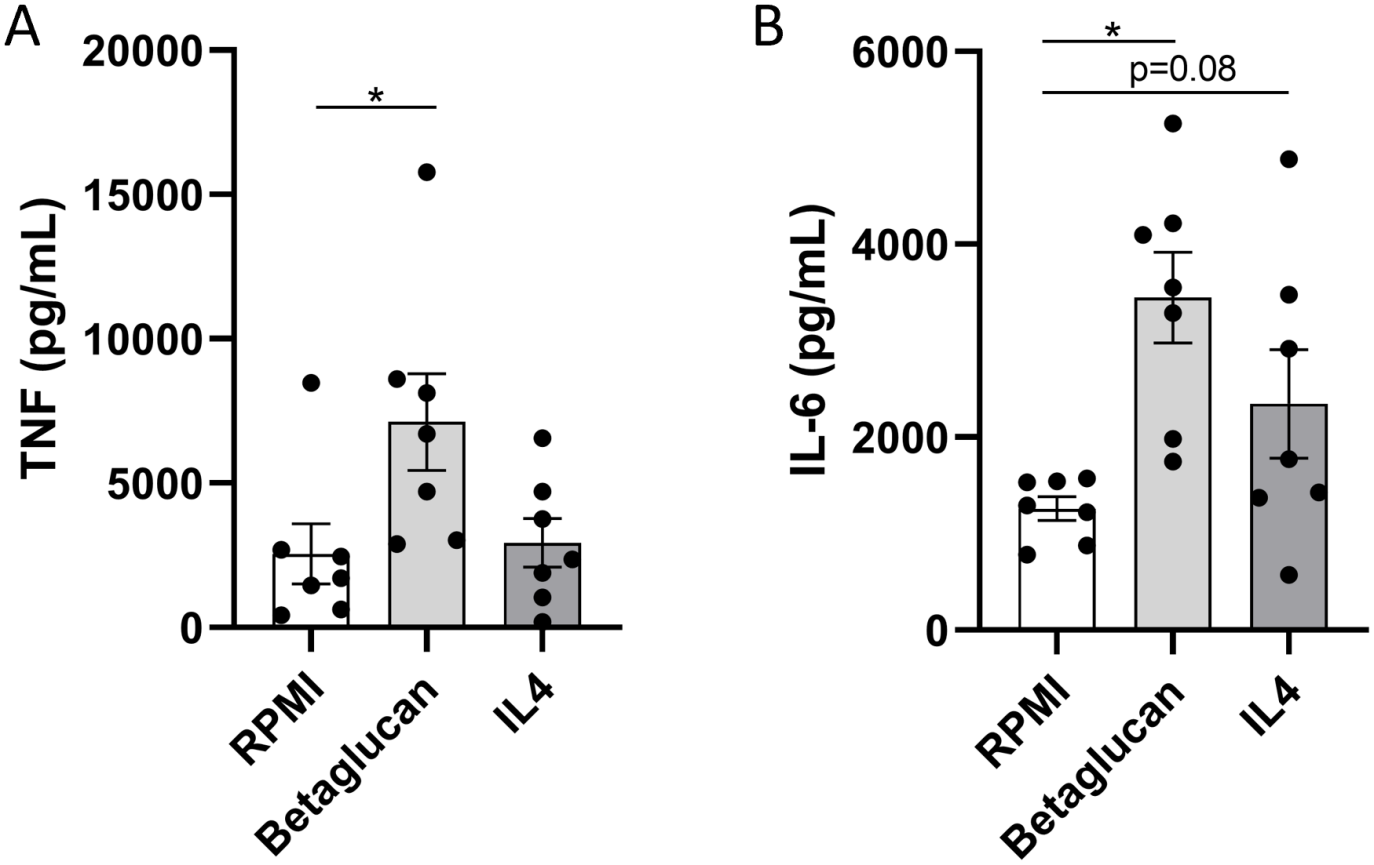
TNF and IL-6 concentrations after restimulation with LPS of β-glucan or IL-4-trained monocytes of colorectal carcinoma patients. RPMI, Roswell Park Memorial Institute; LPS, lipopolysaccharide. *p < 0.05.

To assess whether trained immunity induction can also be achieved in other types of cancer, monocytes from CRC patients were trained and restimulated *ex vivo* (**Figure 2**). Monocytes of CRC patients displayed the capacity to be trained with β-glucan, characterized by increased TNF and IL-6 production upon restimulation compared to untrained controls. In contrast, training with IL-4 did not induce increased cytokine production capacity upon restimulation in monocytes of CRC patients.

### Influence of sex and age on trained immunity induction in NMTC

**Figure 3:**
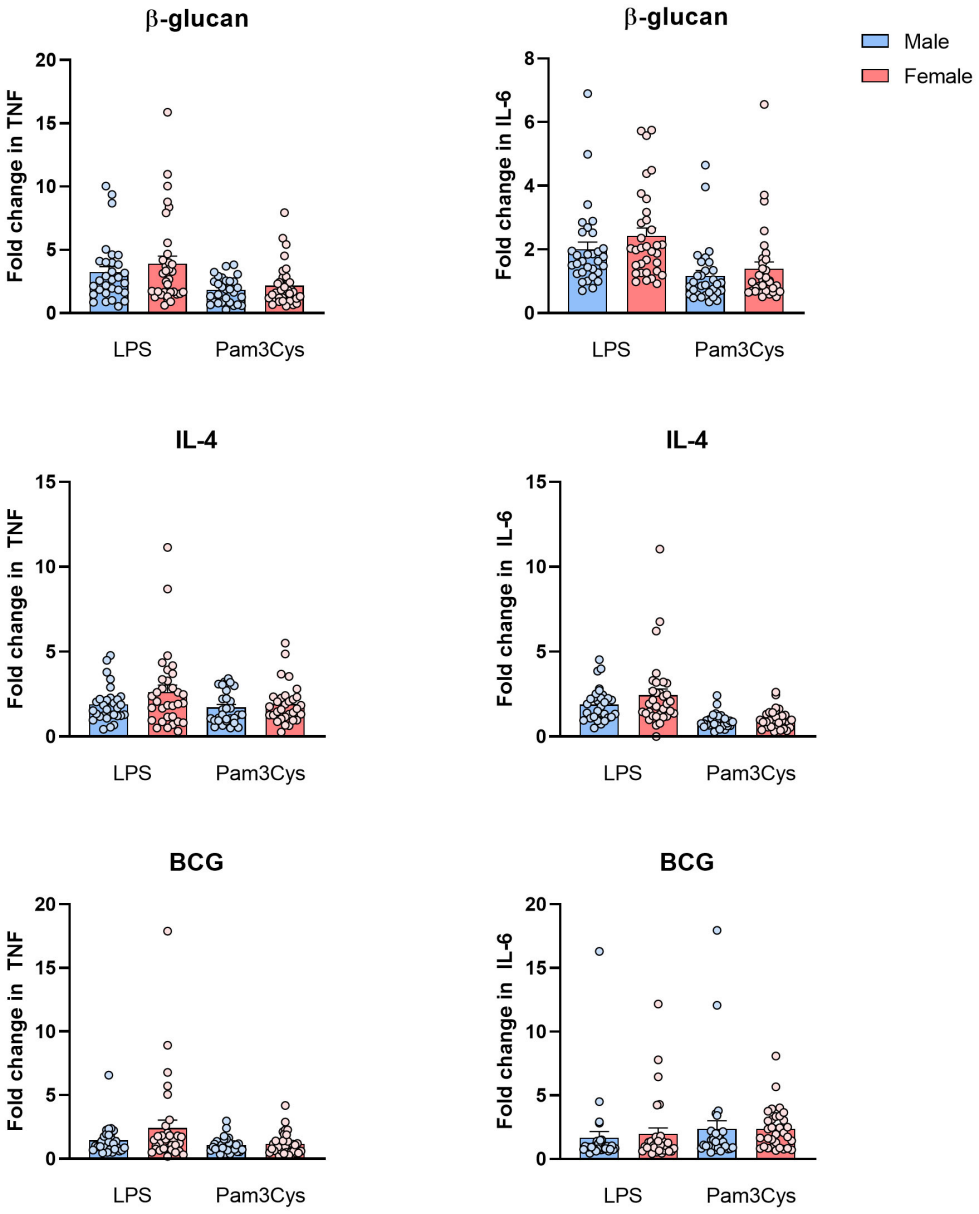
Fold changes (mean ± SEM) in cytokine production after training in male (blue, *n* = 31) and female (red, *n* = 32) participants.

To investigate the effect of sex on trained immunity, fold changes in cytokine production after training were compared between male and female participants (**Figure 3**). No significant differences in fold changes between male and female participants were observed with either of the stimuli.

Furthermore, correlation coefficients between the fold change of cytokine production after training and age were calculated (**Supplementary Table S1**). No statistically significant correlations could be observed between the age of the participants and fold changes in cytokine production after training with the various stimuli.

### Trained immunity induction in different NMTC subtypes

**Figure 4:**
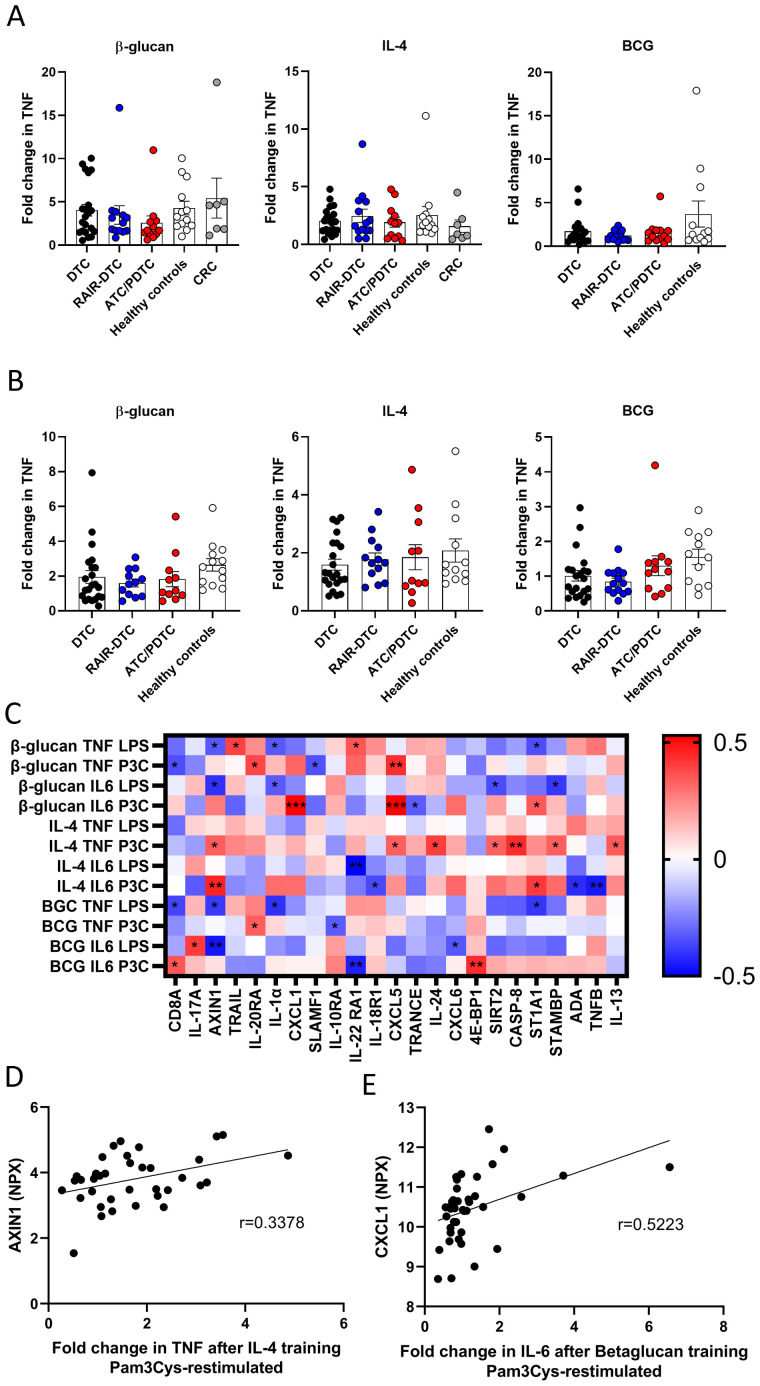
Fold changes in TNF production after restimulation with LPS **(A)** or Pam3Cys **(B)** after training of circulating monocytes from different TC subgroups, healthy controls, and CRC patients. **(C)** Spearman’s correlation coefficients between fold changes in cytokine production after training and normalized protein expression (NPX) values of inflammation-related proteins, measured in plasma using Olink. **(D)** Correlation between NPX of plasma AXIN1 and fold change in TNF production after Pam3Cys restimulation after IL-4 training. **(E)** Correlation between NPX of plasma CXCL1 and fold change in IL-6 production after Pam3Cys restimulation after β-glucan training. CRC, colorectal carcinoma; RPMI, Roswell Park Memorial Institute; DTC, differentiated thyroid carcinoma; RAIR-DTC, radioiodine-refractory differentiated thyroid carcinoma; PDTC, poorly differentiated thyroid carcinoma; ATC, anaplastic thyroid carcinoma; LPS, lipopolysaccharide. *p < 0.05, **p < 0.01, and ***p < 0.001.

We subsequently assessed whether the induction of trained immunity differs in patients with different NMTC subtypes (DTC, RAIR-DTC, and ATC/PDTC) and healthy controls (**Figures 4A, B**, **Supplementary Figure S2**). We observed no differences between the different NMTC subtypes and healthy controls. Notably, training with β-glucan or IL-4 resulted in elevated cytokine production in all subgroups. These results indicate that trained immunity can be induced in circulating monocytes from patients with NMTC regardless of the clinical phenotype, including those with aggressive forms of disease. Interestingly, training with β-glucan induced significantly higher fold changes of IL-6 production upon restimulation in monocytes of CRC patients compared with ATC/PDTC patients (**Supplementary Figure S2**).

### Correlation of trained immunity induction with inflammatory plasma proteins in NMTC

To investigate possible factors that predict the trained immunity response, correlations between inflammation-related proteins, measured in plasma by Olink Proteomics, Uppsala, Sweden, and fold changes in cytokine production after training were assessed in a subset of NMTC patients (*n* = 42: 21 DTC, 10 RAIR-DTC, and 11 ATC/PDTC). All proteins with at least one significant correlation are depicted as a heatmap in **Figure 4C**, with **Figures 4D, E** as examples. Several patterns could be observed: i) chemokines C-X-C motif ligand (CXCL) 1 and 5 were positively correlated with fold changes in cytokine production after Pam3Cys restimulation; ii) axis inhibition protein 1 (AXIN1) was, on the one hand, negatively correlated with cytokine production after LPS restimulation after training with β-glucan or BCG but, on the other hand, was positively associated with cytokine production after Pam3Cys restimulation after training with IL-4; iii) in addition, IL-22 RA1 was negatively associated with IL-6 production after training with IL-4 or BCG; iv) finally, IL-1α plasma concentrations were negatively associated with cytokine production after LPS restimulation after training with β-glucan or BCG. When comparing the different NMTC subgroups, no significant differences were observed in the baseline values of the mentioned inflammatory proteins (**Supplementary Figure S3**). These results indicate that circulating biomarkers are associated with the effectiveness of inducing a trained immunity response.

### Induction of trained immunity in bone marrow progenitors

**Figure 5:**
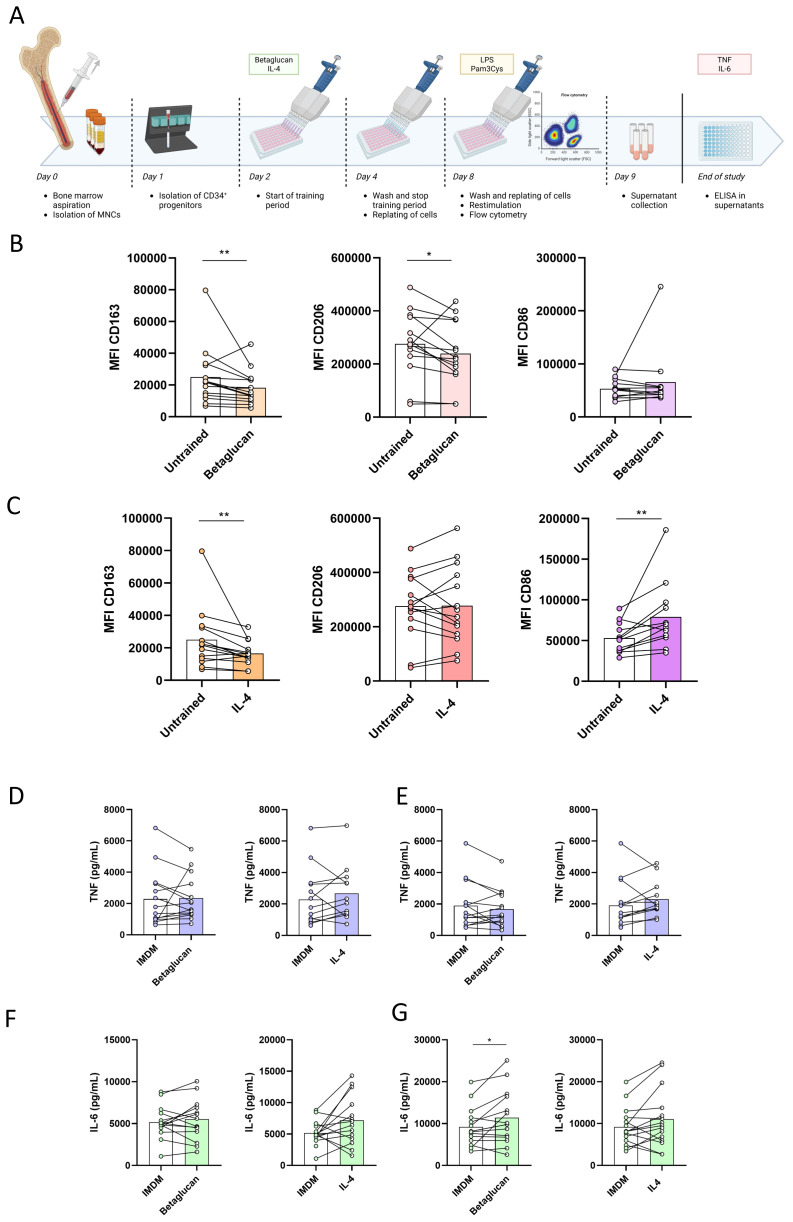
**(A)** Overview of protocol for training of CD34+ progenitors. MFI of C163, CD206 and CD86 on macrophages after training with β-glucan **(B)** or IL-4 **(C)**, compared to untrained controls. **(D)** TNF production after LPS restimulation of trained macrophages, compared to untrained controls. **(E)** TNF production after Pam3Cys restimulation of trained macrophages, compared to untrained controls. **(F)** IL-6 production after LPS restimulation of trained macrophages, compared to untrained controls. **(G)** IL-6 production after Pam3Cys restimulation of trained macrophages, compared to untrained controls. MFI: median fluorescence index, IMDM: Iscove’s Modified Dulbecco’s Medium. *:*p*<0.05 **:*p*<0.01.

To investigate whether trained immunity can be induced in myeloid bone marrow progenitors, a novel methodology was developed for *in vitro* training of human hematopoietic stem and progenitor cells (HSPCs) (**Figure 5A**, and see below Methods). In short, CD34^+^ progenitors were isolated from fresh bone marrow using CD34 microbeads. After 24 hours of resting, cells were stimulated with either β-glucan or IL-4 and differentiated into macrophages. The phenotype of these bone marrow-derived macrophages was assessed using flow cytometry (**Figures 5B, C**). Training of the bone marrow progenitors resulted in macrophages with lower expression of “M2-like” markers CD163 and CD206, while expression of the “M1-like” marker CD86 was either not altered (β-glucan) or increased (IL-4). Likewise, ratios between CD163 or CD206 and CD86 were decreased in trained bone marrow-derived macrophages compared to the untrained controls (**Supplementary Figures S5A, B**). A comparison between NMTC patients and healthy controls showed the same pattern of changes in cell surface marker expression after training (**Supplementary Figures 4**). Training of CD34^+^ bone marrow progenitors did not alter the expression of PD-L1, TLR2, or TLR4 on differentiated macrophages (**Supplementary Figures S5C–E**).

Cytokine production capacity was assessed by restimulating bone marrow-derived macrophages for 24 hours with LPS or Pam3Cys (**Figures 5D–G**). No changes could be observed in cytokine production after restimulation of trained bone marrow-derived macrophages, except for an increased IL-6 production after Pam3Cys restimulation of β-glucan-trained macrophages. To visualize the extent of the plasticity of myeloid cells of individual donors, the fold change in cell surface marker expression on bone marrow-derived macrophages was correlated to the fold change in cytokine production after restimulation with LPS of monocyte-derived macrophages (**Supplementary Figure S6**). Although the statistical power of this analysis was hindered by the limited number of donors of whom both circulating monocytes and bone marrow progenitors were available (*n* = 10), an overall negative correlation was observed between the fold changes, which could indicate that the degree of the plasticity of bone marrow progenitors correlated with the degree of the plasticity of circulating monocytes from the same donor.

## Discussion

Despite the successes of novel immunotherapies targeting T lymphocytes, such as ICIs or chimeric antigen receptor (CAR) T cells, only a minority of patients are responsive to ICIs, while CAR T cells are, so far, only approved in hematological cancers. The limited efficacy of T cell-based immunotherapies in certain cancers may be attributed to an immunosuppressive TME, which results from the presence of inhibitory TAMs. Several approaches have been proposed to target TAMs in cancer, from their complete elimination to reprogramming of their pro-tumorigenic function (4). One of the most promising approaches to reprogram the function of TAMs toward a more anti-tumoral phenotype is through the induction of trained immunity and reprogramming of HSPCs toward a more immunoactive phenotype. While the induction of trained immunity is highly effective in murine models of cancer and can be induced in peripheral myeloid cells from healthy individuals, it is not known whether it can also be achieved in myeloid cells and their progenitors isolated from patients with cancers, in which this phenomenon could potentially be exploited as a treatment strategy.

In the present study, we demonstrate that trained immunity can be induced in myeloid cells from patients with two forms of cancer: NMTC and CRC. As trained immunity needs to be induced *in vivo* in the bone marrow progenitors of myeloid cells to ensure its long-term effects, we developed a new methodology to investigate trained immunity induction in bone marrow progenitor cells, which is more robust and easier to use compared to the existing protocols (30). Subsequently, using this new method, we demonstrate that a trained immunity phenotype can be induced in circulating monocytes and CD34^+^ immune progenitors of patients with NMTC, including those with the most aggressive phenotypes, and CRC, with a similar efficacy as cells from healthy volunteers. The resulting trained myeloid cells have improved responsiveness in terms of cytokine production capacity and express cell surface biomarkers associated with an anti-tumoral phenotype. This provides the first step for an emerging possibility of *ex vivo* training of myeloid cells or immune cell progenitors, which could subsequently be used in the future as cell-based therapy in patients with cancer. Future studies are warranted to explore the effects of these trained cells in experimental cancer models and later in clinical trials.

The results of the present study are important at several levels. First, we demonstrate that trained immunity can be induced in myeloid cells, both in circulating monocytes from NMTC patients and CRC patients, and in myeloid bone marrow progenitors from NMTC patients. Using the well-established *ex vivo* trained immunity protocol, we observed an increased production of TNF and IL-6, well-established read-outs of trained immunity, upon restimulation of monocytes after they were trained with β-glucan or IL-4. Second, the efficacy of trained immunity induction was similar in patients with NMTC regardless of their subtype and in healthy controls. This indicates that in patients with NMTC, the monocytes retain their functional plasticity and could therefore be amenable to therapeutic interventions targeting their response to trained immunity agonists, opening the door for trained immunity-based immunotherapy. Unsurprisingly, there was heterogeneity in the response to the different training stimuli, which can be explained by the different mechanisms activated by these stimuli. For example, IL-4 was able to induce trained immunity in NMTC, but not in CRC patients. Conversely, β-glucan induces higher fold changes of IL-6 production upon restimulation with LPS in CRC patients compared to ATC/PDTC patients. The source of these differences should be investigated in future studies, also considering other (epi)genetic and environmental factors that influence the efficacy of trained immunity (31, 32). In this respect, it is likely that different tumors release distinct mediators (including cytokines, growth factors, and metabolites) that reprogram progenitor and mature myeloid cells in unique ways, explaining these differences. Nonetheless, the robust response to β-glucan and IL-4 training provides the necessary proof-of-concept that myeloid cells from NMTC patients can be reprogrammed through trained immunity. Third, since trained immunity could be induced in monocytes from both NMTC and CRC patients, we envisage that our results could be generalized to other types of cancer as well. This conclusion is supported by the anti-tumor effects of BCG in bladder cancer, in which the induction of trained immunity has been shown to be an important mechanism of action (28, 29). We hypothesize that trained immunity-based cell therapy represents a promising novel type of immunotherapeutic approach in cancer patients in general, either as re-administration of *ex vivo* trained autologous myeloid cells or by *in vivo* administration of trained immunity-inducing agents, and future studies to assess this hypothesis should be pursued.

There are also remaining questions to be answered. In this study, we used increased TNF and IL-6 production as well-established read-outs of trained immunity. Importantly, however, the *in vivo* induction of trained immunity is more complex and involves the production of numerous other cytokines, as well as the modulation of additional cellular functions, which could explain why the effect of trained immunity is not limited to the potential effects of these pleiotropic cytokines (33, 34). A more comprehensive assessment of myeloid cells derived from trained immune progenitors should be initiated in future studies. Likewise, it remains to be assessed whether the cytokine production response upon *ex vivo* training can also predict the *in vivo* training response.

To predict how effectively the innate immune cells of a patient can be reprogrammed, biomarkers are needed. For this reason, we assessed correlations between fold changes of cytokine production after restimulation and several inflammation-related proteins that were measured in plasma using a proteomics approach. We observed several significant correlations. For example, AXIN1 was correlated negatively to fold change in cytokine production after restimulation with LPS, on the one hand, but, on the other hand, correlated positively to fold change after restimulation with Pam3Cys following IL-4 training. This discrepancy can be explained by the fact that LPS and Pam3Cys act through different receptors. AXIN1 is involved in several signaling cascades, among which are the WNT/β-catenin, Hippo, AMPK, and mTOR pathways (35). Interestingly, a recent study from our group showed that AXIN1 was also negatively correlated with fold change in TNF production upon stimulation with SARS-CoV-2 after influenza vaccination (36). Furthermore, the chemoattractants CXCL1 and CXCL5 were strongly positively correlated with fold change in cytokine production after restimulation. These findings underline interindividual differences in susceptibility to trained immunity and the importance of biomarkers that can predict successful training. It should be noted that only a selected panel of inflammation-related proteins was used for this analysis. Future studies with larger cohorts and larger panels of proteins and cytokines are needed to expand and validate these findings.

Previous studies have shown that the effects of trained immunity can take place in bone marrow progenitors, which are likely responsible for its durable effects (19, 20). Therefore, we designed a new experimental protocol for *ex vivo* reprogramming and training of bone marrow progenitors: we demonstrated that the induction of a trained immunity anti-tumor phenotype is achievable in NMTC patients. For this, we exposed bone marrow progenitors to either β-glucan or IL-4, and we differentiated these trained progenitors into macrophages. Interestingly, macrophages differentiated from the trained progenitors showed significantly lower expressions of the markers CD163 and CD206. High expression of the scavenger receptor CD163 and the mannose receptor CD206 on macrophages has been linked to poor outcomes in several cancers, among which are NMTC, breast cancer, melanoma, gastric cancer, liver cancer, and oral squamous cell cancer (37–42). Moreover, the expression of CD86 increased after training with IL-4 and was unaltered after training with β-glucan. CD86 expression on TAMs has been linked with improved survival of patients with CRC and liver cancer (43, 44). The observed decreased expression of both CD163 and CD206 and the increased or stable expression of CD86 in macrophages derived from trained bone marrow progenitors emphasize the oncological therapeutic potential of trained immunity.

One limitation of this study is the fact that we could not obtain bone marrow from patients with ATC or PDTC due to logistical challenges, including the rapid presentation of cases and the fact that most of these patients are not eligible for surgery. Therefore, we should be cautious when generalizing our data to this patient category, and future studies should prioritize including these patients for similar analyses. However, the fact that, in our series, the capacity to mount cytokine production of trained circulating monocytes did not differ between patients with ATC/PDTC and patients with DTC indicates that the functional plasticity of the innate immune cells and thus their capacity to respond to training is retained even in the most aggressive types of NMTC. This study provides robust proof that reprogramming of both circulating monocytes and bone marrow progenitors of NMTC patients is feasible and promising as a therapeutic strategy. Indeed, several experimental murine studies have shown that transplantation of bone marrow progenitors from trained mice or suppletion with trained immune cells has potent anti-tumoral effects (23–27). Nonetheless, future research should investigate the anti-tumoral effects of the *in vivo* trained immunity hypothesis in experimental NMTC models. Moreover, research should be extended toward assessing the potential of trained macrophages to stimulate other immune cells like cytotoxic T cells and natural killer cells, as the interactions between the innate and adaptive immune system shape the TME. An additional limitation is that it was inevitable to differentiate the bone marrow progenitors using a medium that was supplemented with human pooled serum and several growth factors. Since these growth factors could also interfere with the functional programming of the macrophages (macrophage colony-stimulating factor, for example, programs macrophages toward an M2-like phenotype), we cannot rule out a dampening outcome of these growth factors on the effects of training (45). Moreover, long-term induction of a trained immunity phenotype across multiple generations of HSPCs also needs to be investigated in future *in vivo* studies.

In conclusion, this is the first study to investigate whether trained immunity can be induced *ex vivo* in monocytes and immune cell progenitors from patients with cancer with the use of NMTC and CRC as relevant malignancies sharing the tumor-related innate immune responses as an important pathogenetic mechanism. Both circulating monocytes and bone marrow progenitors could be trained, independent of age, sex, or cancer subtype. Functional and phenotypical changes were observed in macrophages after inducing trained immunity *ex vivo*, which could open avenues to further explore the potential of trained immunity-based cell therapy as a novel immunotherapy in cancer. Further exploration of the therapeutic potential of *ex vivo* trained innate immune cells could provide a new direction for the management of advanced NMTC as well as other malignancies that are refractory to conventional treatments. Future studies, including larger cohorts in which patients with aggressive forms of cancer are more represented, are essential in order to gain the mechanistic insights required to translate these initial findings into potential clinical applications.

## Methods

### Patient selection

Peripheral blood was collected from healthy controls, NMTC patients, and CRC patients between September 2022 and December 2024. NMTC and CRC patients were either newly diagnosed (these cases were included before they underwent surgery) or undergoing outpatient clinic follow-up at the Radboud University Medical Center, Nijmegen, the Netherlands. Bone marrow was collected from the patients who underwent surgery. In addition, leftover bone marrow from healthy stem cell donors was obtained through the Hematology Department of the Radboud University Medical Center. The NMTC patients that were undergoing follow-up all had aggressive subtypes, defined as ATC, PDTC, or RAIR-DTC, classified as follows: 1 = negative RAI uptake, 2 = RAI uptake in some but not all metastases, or 3 = disease progression 6–12 months after RAI treatment (46). The criteria for exclusion were as follows: age below 18 years, mental incompetence, pregnancy or breastfeeding, inflammatory or infectious comorbidities, treatment with immunomodulating medication, other active malignancies, self-reported alcohol consumption of >21 units per week, or surgery within the previous 4 months.

### Study approval

The study was approved by the local Ethics Committee (Commissie Mensgebonden Onderzoek Arnhem-Nijmegen: 2021-13380 for newly diagnosed NMTC patients and all CRC patients, 2022-16025 for patients with aggressive NMTC undergoing follow-up, NL84281.091.23 for healthy controls donating blood, and CMO 2013-064 for healthy controls donating bone marrow). The study was registered at ClinicalTrials.gov (NCT05280379). All donors provided written informed consent, and all study procedures were conducted according to the principles of the Declaration of Helsinki.

### Blood sampling and bone marrow aspiration

Blood was collected by venipuncture in ethylenediaminetetraacetic acid (EDTA) collection tubes. Plasma was obtained from blood by centrifugation and stored at −80°C until further use. Bone marrow was collected in patients undergoing surgery immediately after induction of general anesthesia and tracheal intubation and before intravenous administration of any glucocorticoids. The bone marrow from thyroid carcinoma patients was drawn from the posterior iliac crest in syringes containing sodium heparin (end concentration 35 IU/mL) by an experienced physician assistant. For the healthy controls that donated bone marrow, an anticoagulation mix was used containing 500 mL ACD-A (Fresenius, Bad Homburg vor der Höhe, Germany) + 25.000 IU heparin LEO (Baxter, Deerfield, IL, USA). One milliliter of this mix was then added to 9 mL of aspirated bone marrow.

### Isolation of PBMCs and monocytes

From the EDTA blood, peripheral blood mononuclear cells (PBMCs) were isolated via density gradient centrifugation using Ficoll-Paque (VWR, Radnor, PA, USA) and SepMate tubes (Stemcell Technologies, Vancouver, BC, Canada). PBMCs were washed twice in cold phosphate-buffered saline (PBS; Gibco, Waltham, MA, USA) and resuspended in medium (Roswell Park Memorial Institute (RPMI), Gibco, supplemented with gentamycin 50 µg/mL, pyruvate 1 mM, and GlutaMAX 2 mM). Cells were counted using a Sysmex-XN 450 hematology analyzer (Sysmex, Kobe, Japan). Monocytes were isolated from the PBMC fraction via negative selection using microbeads (Pan Monocyte isolation kit, Miltenyi Biotec, Bergisch Gladbach, Germany) according to the manufacturer’s instructions.

### Induction of trained immunity in monocytes

Training of monocytes was performed in 96-well flat-bottom plates as described previously (47). Per well, 1.5 × 10^5^ adherent monocytes were stimulated for 24 hours with β-glucan (10 µg/mL, AB Biotek, St. Louis, MOS, USA), IL-4 (25 ng/mL, R&D Systems, Minneapolis, MN, USA), BCG (5 µg/mL, AJ Vaccines, Copenhagen, Denmark), or RPMI medium as a negative control. After 24 hours of training, wells were washed, and fresh RPMI medium supplemented with 10% human pooled serum was added. After 2 days of resting, the medium was replaced with fresh medium. On day 6, cells were restimulated with *E. coli*-derived LPS (10 ng/mL, *E. coli* 055:B5, Sigma-Aldrich, St. Louis, MO, USA), Pam3Cys-Ser-(Lys)4 (Pam3Cys, 10 µg/mL, EMC, Tübingen, Germany), or RPMI medium as a negative control. All stimulations and restimulations were performed in duplicate. After 24 hours of restimulation, supernatants were collected and stored at −20°C until further use.

### Isolation of mononuclear cells and CD34^+^ progenitors

The bone marrow aspirate was filtered and washed with PBS. Mononuclear cells (MNCs) were isolated from the bone marrow via density gradient centrifugation using Ficoll-Paque. MNCs were washed twice and then resuspended in Iscove’s Modified Dulbecco’s Medium (IMDM; Gibco, supplemented with gentamycin 50 µg/mL and 10% human pooled serum). MNCs were kept in a fridge overnight. The next day, CD34^+^ progenitors were isolated from the MNC fraction using a CD34 MicroBead kit (Miltenyi Biotec), according to the manufacturer’s protocol. CD34^+^ progenitors were then resuspended in IMDM and counted manually using Trypan Blue (Merck, Darmstadt, Germany). The purity was assessed on a CytoFLEX flow cytometer (Beckman Coulter, Fullerton, CA, USA).

### Induction of trained immunity in CD34^+^ progenitors

Per well, 0.5 × 10^5^ CD34^+^ progenitors were plated on a 48-well plate. To proliferate and differentiate the progenitors, IMDM supplemented with 20% human pooled serum and several growth factors (stem cell factor 25 ng/mL, macrophage colony-stimulating factor 30 ng/mL, interleukin-3 30 ng/mL, and FMS-like tyrosine kinase 3 ligand 30 ng/mL (all from Miltenyi Biotec)) was used. After 24 hours of incubation (37°C, 5% CO_2_), medium containing the trained immunity-inducing stimuli was added to the cells: β-glucan 1 µg/mL, IL-4 50 ng/mL, or medium as a negative control. After 48 hours, the training was stopped by discarding the medium and detaching the cells using cold PBS supplemented with 10 mM EDTA. Detached cells were washed in PBS, resuspended in IMDM, and counted manually using Trypan Blue; 1.0 × 10^5^ cells per well were then plated on a 48-well plate in IMDM supplemented with human pooled serum and the growth factors mentioned before to further differentiate the cells into macrophages. After an additional 4 days of resting, the cells were detached again as described before and counted manually using Trypan Blue.

For restimulation, macrophages were plated on a 96-well flat-bottom plate, 0.75 × 10^5^ per well. Macrophages were stimulated in duplicate with LPS 10 ng/mL, Pam3Cys 10 µg/mL, or medium as a negative control. After 24 hours of stimulation, supernatants were collected and stored at −20°C until further use.

### Cytokine assays

Concentrations of IL-6 and tumor necrosis factor (TNF) were determined in supernatants using commercial enzyme-linked immunosorbent assay (ELISA) kits (R&D Systems) according to the manufacturer’s instructions. All supernatants belonging to one donor were measured on the same plate.

### Flow cytometry

To assess the phenotype of bone marrow-derived macrophages, 1.0 × 10^5^ cells per training condition were first stained with Fixed Viability Stain 620 (live/dead stain, BD Biosciences, Franklin Lakes, NJ, USA). In parallel, 1.0 × 10^4^ cells per condition were used as unstained controls. After 15 minutes of incubation, cells were washed with PBA-EDTA (PBS supplemented with 1% bovine serum albumin (BSA) and 2 mM EDTA). Thereafter, cells were resuspended in PBA-EDTA, and Fc receptors were blocked using TruStain FcX (BioLegend, San Diego, CA, USA) for 5 minutes. Then, cells were stained using a panel consisting of the following antibodies: CD14-FITC, CD206-PE, CD45-PerCp, CD86-PC7, CD163-APC, CD3-APC-Cy7, CD20-APC-Cy7, CD19-APC-Cy7, CD56-APC-Cy7, CD11b-BV421, and CD16-BV510 (all from BioLegend). In parallel, a second set of 1.0 × 10^5^ macrophages was stained using a panel consisting of the following antibodies: TLR2-FITC, TLR4-PE, CD45-PerCp, PDL1-APC, CD3-APC-Cy7, CD20-APC-Cy7, CD19-APC-Cy7, CD56-APC-Cy7, and CD11b-BV421 (all from BioLegend). After 30 minutes of staining at room temperature, both stained and unstained samples were measured on a CytoFLEX flow cytometer (Beckman Coulter). Data were analyzed using FlowJo (v10.8.1). Representative gating strategy is detailed in **Supplementary Figure S7**.

### Proteomics

Circulating inflammatory markers were analyzed in EDTA plasma from a subset of NMTC patients using the commercial Target 96 Inflammation panel of Olink Proteomics, according to the manufacturer’s instructions. This panel was used to measure 92 inflammation-related proteins by multiplex proximity extension assays, as quantified by real-time PCR.

### Statistics

Statistical analyses were performed using Prism GraphPad (version 10.1.2). Baseline characteristics are presented as *n* or as median and IQR. Fold changes of training were calculated by dividing the cytokine concentration after restimulation of the trained cells by that of the cells that were trained with the medium control (untrained) of the same donor. Categorical variables were compared using Fisher’s exact test. Continuous variables were compared using the Mann–Whitney *U* test in case of non-paired samples or the Wilcoxon signed-rank test in case of paired samples. Correlations were assessed by calculating Spearman’s correlation coefficients. *p*-Values <0.05 were considered statistically significant.

## Data Availability

The raw data supporting the conclusions of this article will be made available by the authors, without undue reservation.
