# Supplementary material for: Induction of trained immunity in myeloid cells and their progenitors from thyroid cancer patients
Source: Front Immunol. 2025 Dec 17;16:1706496. doi: 10.3389/fimmu.2025.1706496 (PMC12838320; doi:10.3389/fimmu.2025.1706496)

Supplemental material

Supplemental Table S1: Spearman's correlation coefficients between fold change of cytokine production after training and age.

Cytokine	Training stimulus	Restimulation	*r* coefficient	*p-value*
TNF	β-glucan	LPS	-0.1255	0.343
		Pam3Cys	-0.0483	0.719
	IL-4	LPS	-0.1433	0.283
		Pam3Cys	-0.0461	0.733
	BCG	LPS	-0.0513	0.705
		Pam3Cys	-0.1369	0.306
IL-6	β-glucan	LPS	-0.2526	0.054
		Pam3Cys	-0.1704	0.197
	IL-4	LPS	-0.2018	0.125
		Pam3Cys	0.0389	0.776
	BCG	LPS	-0.2128	0.112
		Pam3Cys	-0.2311	0.081

TNF: tumor necrosis factor, IL: interleukin, BCG: *Bacillus Calmette-Guérin*, LPS: lipopolysaccharide

Supplemental Figure S1: Flowchart with assessment for eligibility, reasons for exclusions and numbers of included healthy controls and patients.

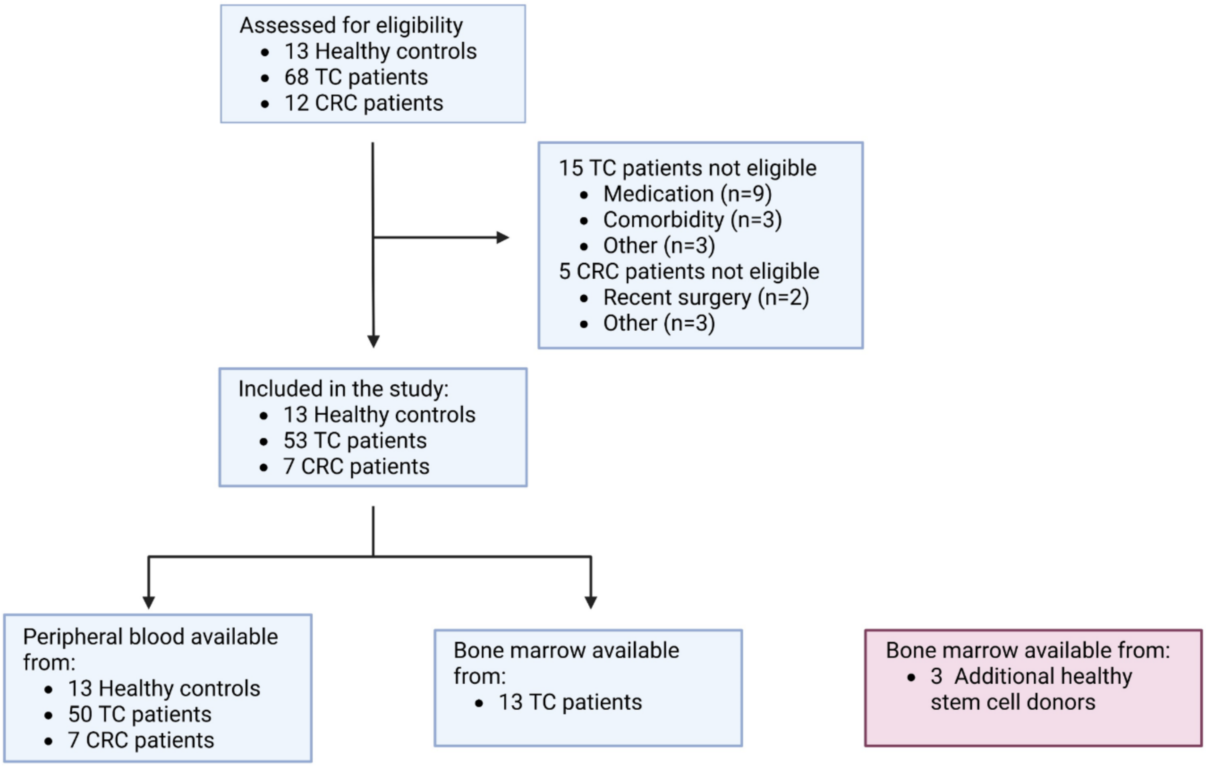

TC: Thyroid carcinoma, CRC: colorectal carcinoma.

Supplemental Figure S2: Fold changes in IL-6 production after restimulation with LPS (A) or Pam3Cys (B) after training of circulating monocytes from different thyroid carcinoma subtypes, healthy controls and CRC patients.

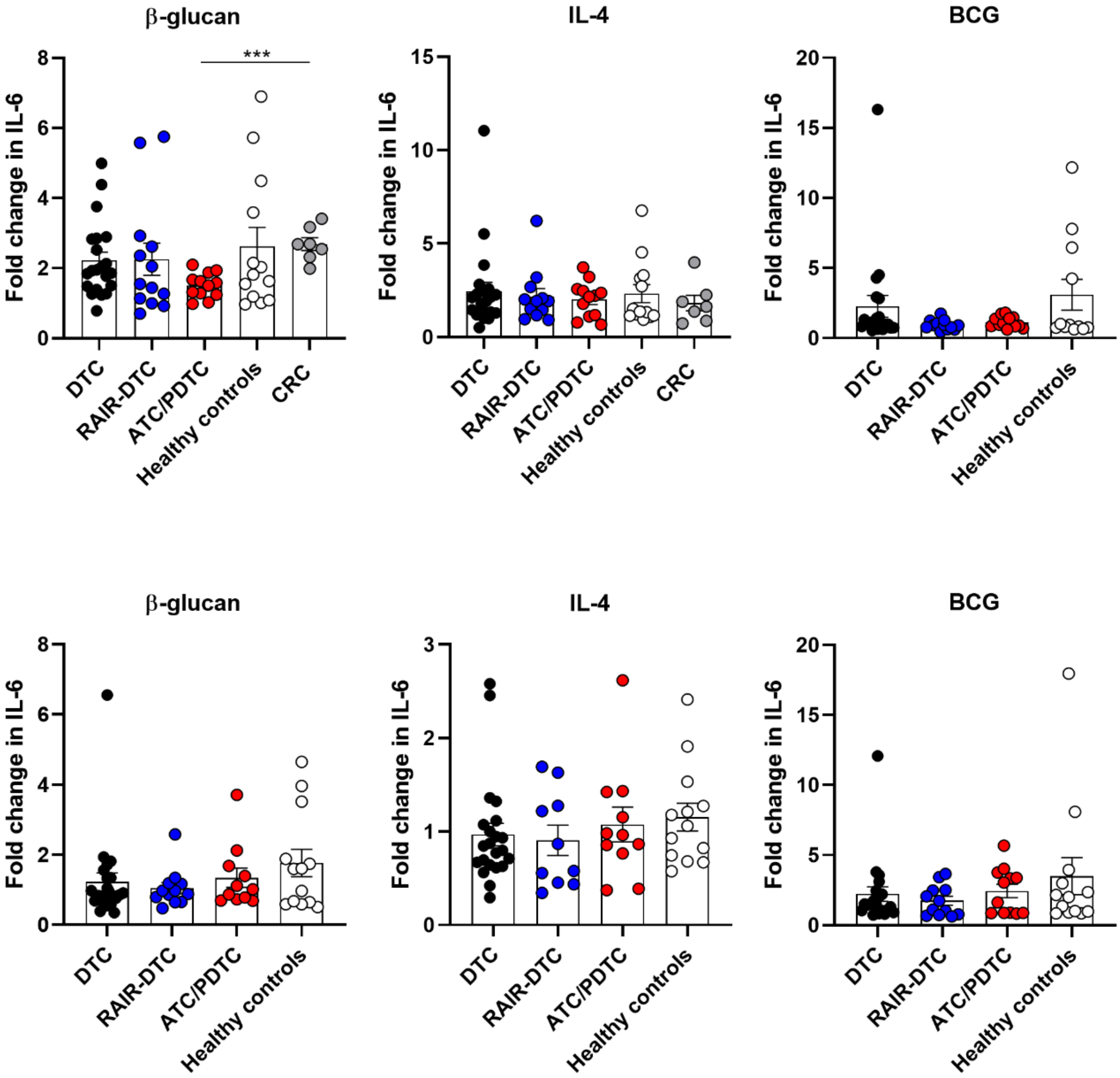

CRC: colorectal carcinoma, RPMI: Roswell Park Memorial Institute, DTC: differentiated thyroid carcinoma, RAIR-DTC: radioiodine-refractory differentiated thyroid carcioma, PDTC: poorly differentiated thyroid carcinoma, ATC: anaplastic thyroid carcinoma. ***: $p<0.001$

Supplemental Figure S3: Comparison of baseline NPX values of inflammatory proteins AXIN1, IL22 RA1, CXCL1, CXCL5, and IL-1α between different thyroid carcinoma subtypes, showing no significant differences.

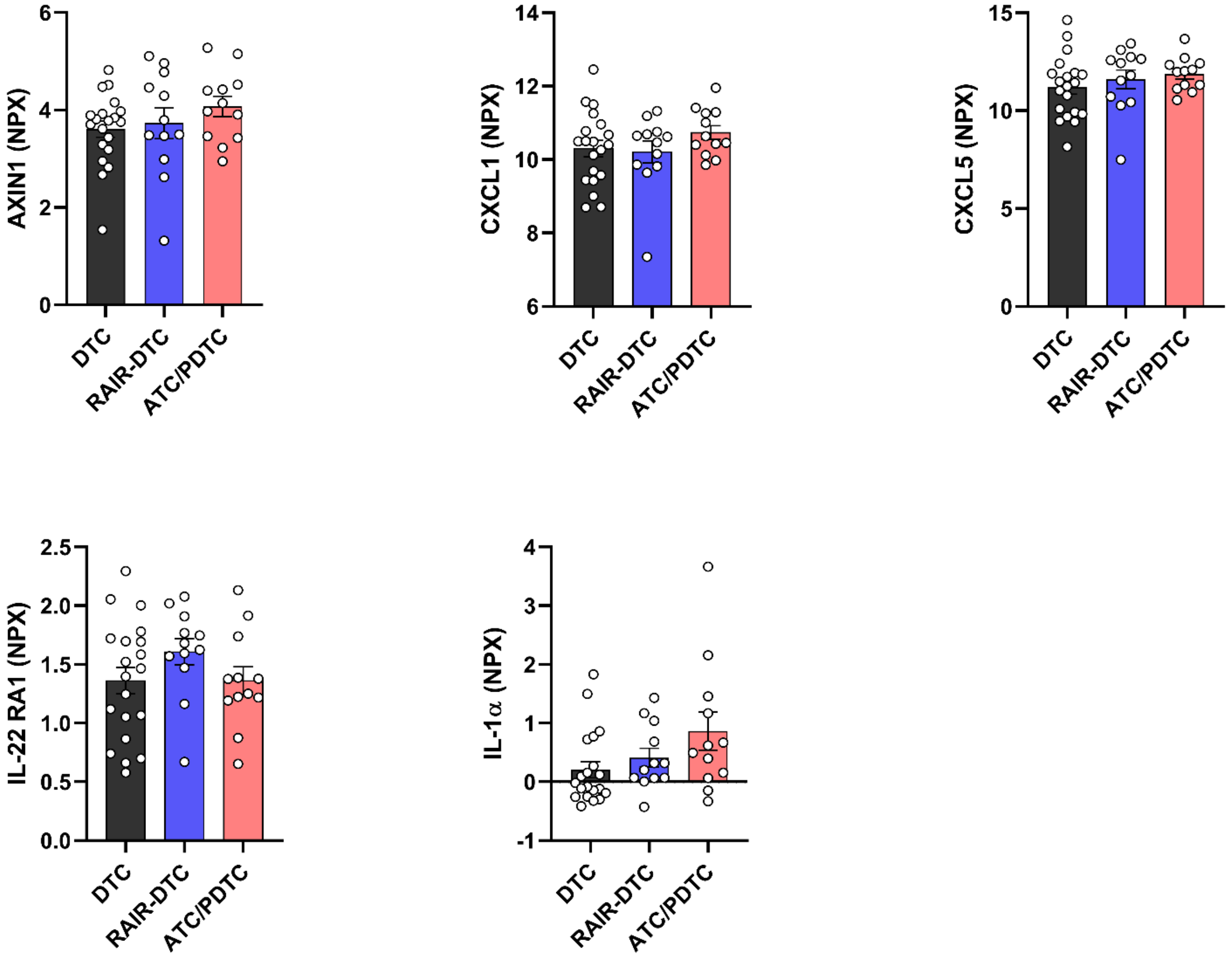

NPX: normalized protein expression, DTC: differentiated thyroid carcinoma, RAIR-DTC: radioiodine-refractory differentiated thyroid carcinoma, PDTC: poorly differentiated thyroid carcinoma, ATC: anaplastic thyroid carcinoma.

Supplemental Figure S4: Median expression of cell surface markers CD163, CD206 and CD86 on trained bone marrow derived macrophages compared to untrained macrophages, for NMTC patients (n=11) and healthy controls (n=3).

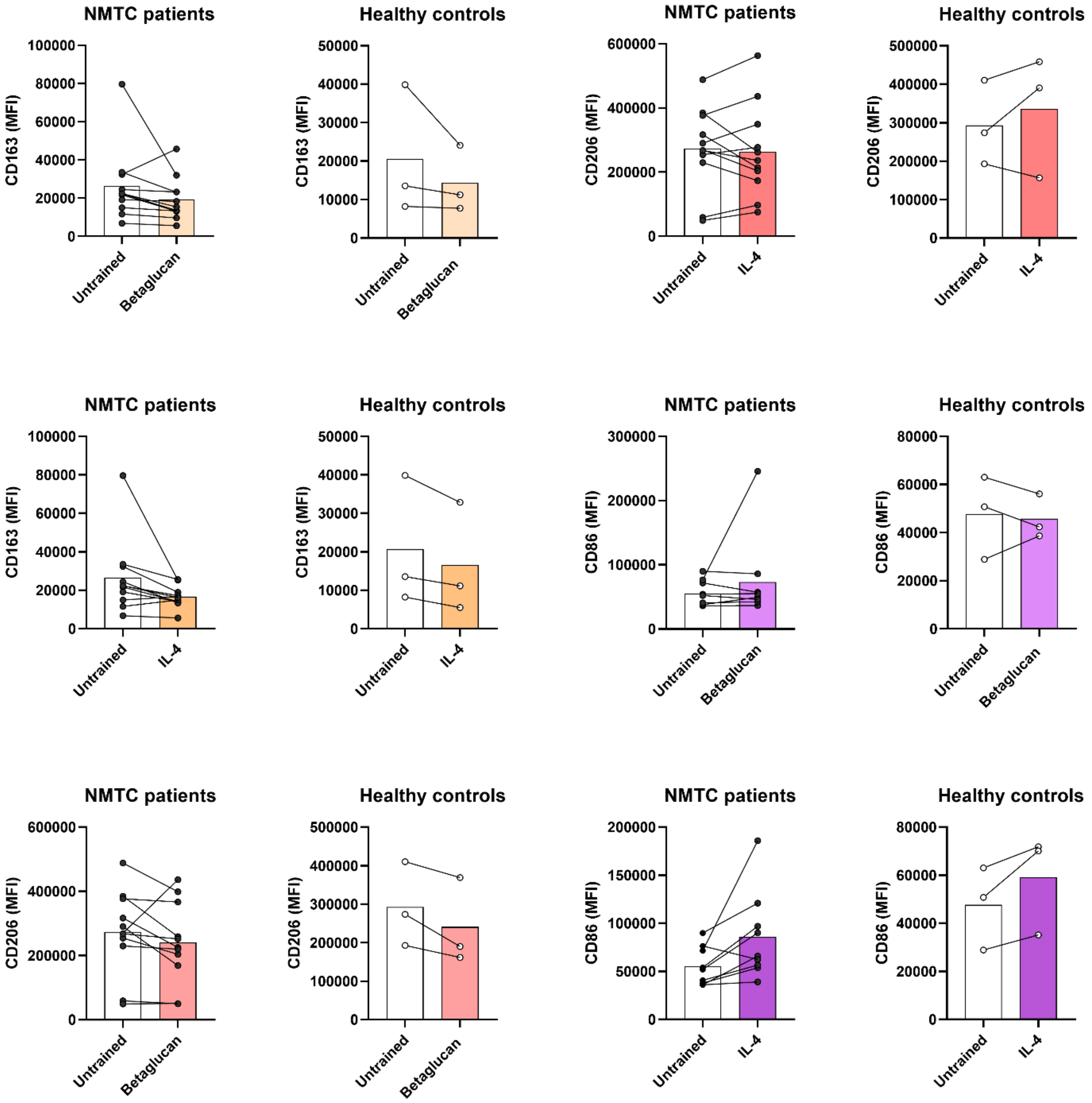

MFI: median fluorescence index.

Supplemental Figure S5: A: Ratio of CD163/CD86 on trained bone marrow derived macrophages compared to controls. B: Ratio of CD206/CD86 on trained bone marrow derived macrophages compared to controls. MFIs of PD-L1 (C), TLR2 (D) and TLR4 (E) on bone marrow derived macrophages after training, compared to controls.

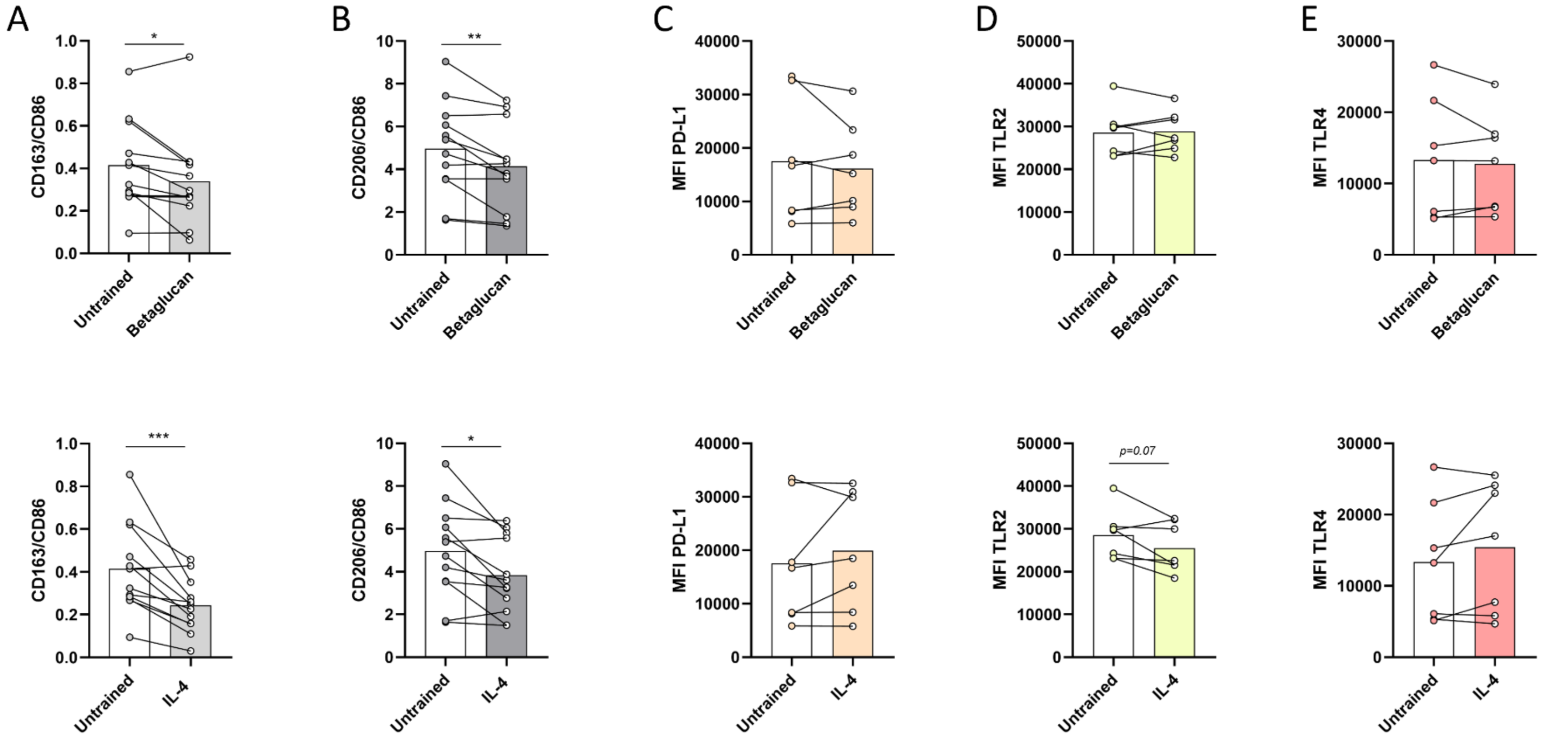

MFI: median fluorescence index. *: p<0.05 **: p<0.01 ***: p<0.001

Supplemental Figure S6. Correlations between fold change in cell surface marker expression on bone marrow-derived macrophages after training with betaglucan and fold change in cytokine production after restimulation with LPS of monocyte-derived macrophages of the same donor.

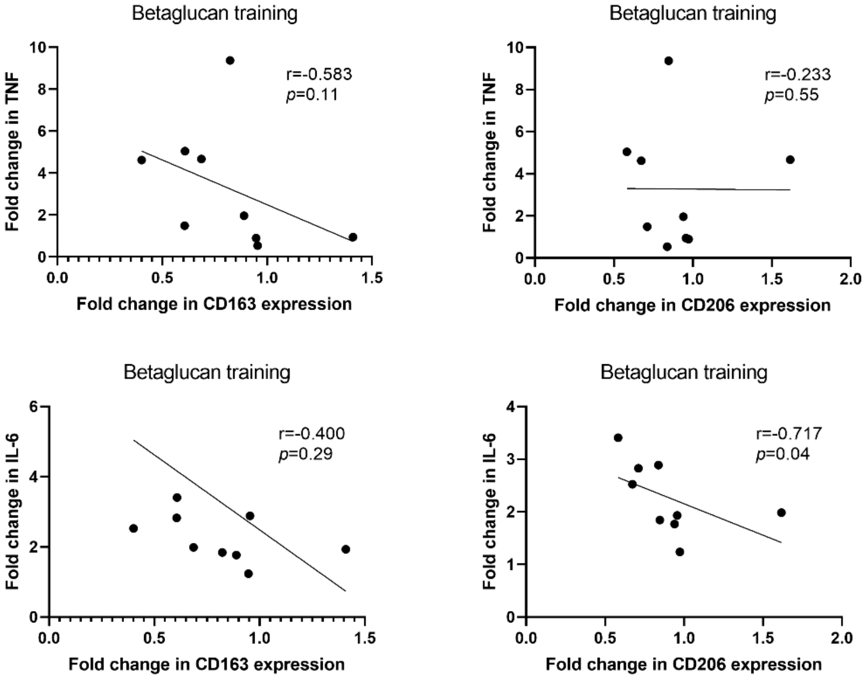

Supplemental Figure S7: Gating strategy for gating bone marrow derived macrophages.

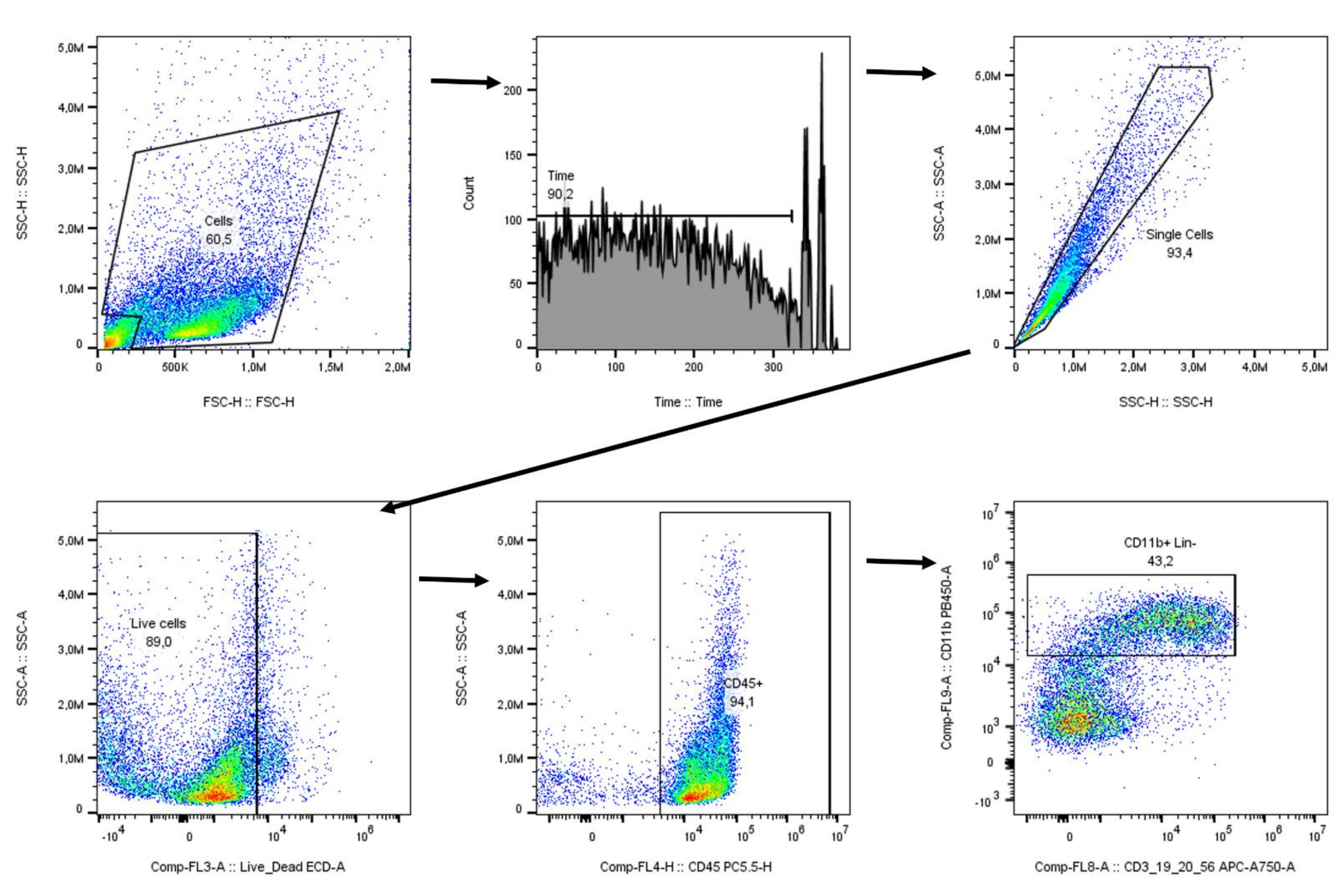

Supplement: Supplementary file 1 [file DataSheet1.pdf]
